# Osteoporotic vertebral compression fractures caused by Cushing’s syndrome in young women: case report and literature review

**DOI:** 10.1186/s12891-023-06253-9

**Published:** 2023-03-06

**Authors:** Jie Cheng, Songli Ju, Zihan Zhang

**Affiliations:** Department of Orthopedic Surgery, Affiliated Hospital of Zunyi Medical University, 149 Dalian Road, Huichuan District, Zunyi, 563000 Guizhou China

**Keywords:** Case report, Osteoporosis vertebral compression fracture, Cushing’s syndrome, Treatment, Anti-osteoporosis

## Abstract

**Background:**

Cushing’s syndrome is known as an important cause of secondary osteoporosis, characterized by reduction of bone mineral density and potential occurrence of fragility fractures before diagnosis in young population. Therefore, for young patients with fragility fractures, especially in young women, more attention should be paid on glucocorticoid excess caused by Cushing’s syndrome, due to relatively higher rate of misdiagnosis, distinct pathological characteristics and different treatment strategies compared with violent fractures and primary osteoporosis related fractures.

**Case presentation:**

We presented an unusual case of a 26-year-old woman with multiple vertebral compression fractures and pelvis fractures, subsequently diagnosed as Cushing’s syndrome. On admission, the radiographic results showed fresh second lumbar vertebra fracture, and old fourth lumbar vertebra and pelvic fractures. The dual energy X-ray absorptiometry of lumbar spine revealed marked osteoporosis, and her plasm cortisol was extremely high. Then, Cushing’s syndrome, caused by left adrenal adenoma, was diagnosed by further endocrinological and radiographic examinations. After receiving left adrenalectomy, her plasma ACTH and cortisol values returned to normal level. In term of OVCF, we adopted conservative treatments, including pain management, brace treatment, and anti-osteoporosis measures. Three months after discharge, the patient’s low back pain was in complete remission without new onset of pain, and returned to normal life and work. Furthermore, we reviewed the literatures on advancements in the treatment of OVCF caused by Cushing’s syndrome, and based on our experiences, proposed some additional perspectives to guide treatment.

**Conclusion:**

In term of OVCF secondary to Cushing’s syndrome without neurological damage, we prefer systematic conservative treatments, including pain management, brace treatment, and anti-osteoporosis measures, to surgical treatment. Among them, anti-osteoporosis treatment has the highest priority because of the reversibility of osteoporosis caused by Cushing’s syndrome.

## Background

Osteoporotic vertebral compression fracture (OVCF) is the third most common type of fragility fracture worldwide in men 50 years or older and postmenopausal women with no more than moderate trauma [1], which can lead to persistent pain, spinal deformity, height loss, depression, poor quality of life, and even death. Cushing’s syndrome (GS) is known as an important etiology of secondary osteoporosis. It is characterized by reduction of bone mineral density (BMD) and potential occurrence of fragility fractures before diagnosis in children or young women [2]. Therefore, for young patients with fragility fracture without history of glucocorticoid abuse, especially in young women, more attention should be paid on glucocorticoid excess caused by Cushing’s syndrome, due to relatively higher rate of misdiagnosis, distinct pathological characteristics and different treatment options compared with violent fractures and primary osteoporosis related fractures. We described a case that lumbar vertebrae compression fracture caused by Cushing’s syndrome diagnosed in a 26-year-old woman who visited our hospital due to repetitive low back pain, and then, literature review was conducted to summarize the advancements of OVCF related to Cushing’s syndrome.

## Case presentation

**Fig. 1:**
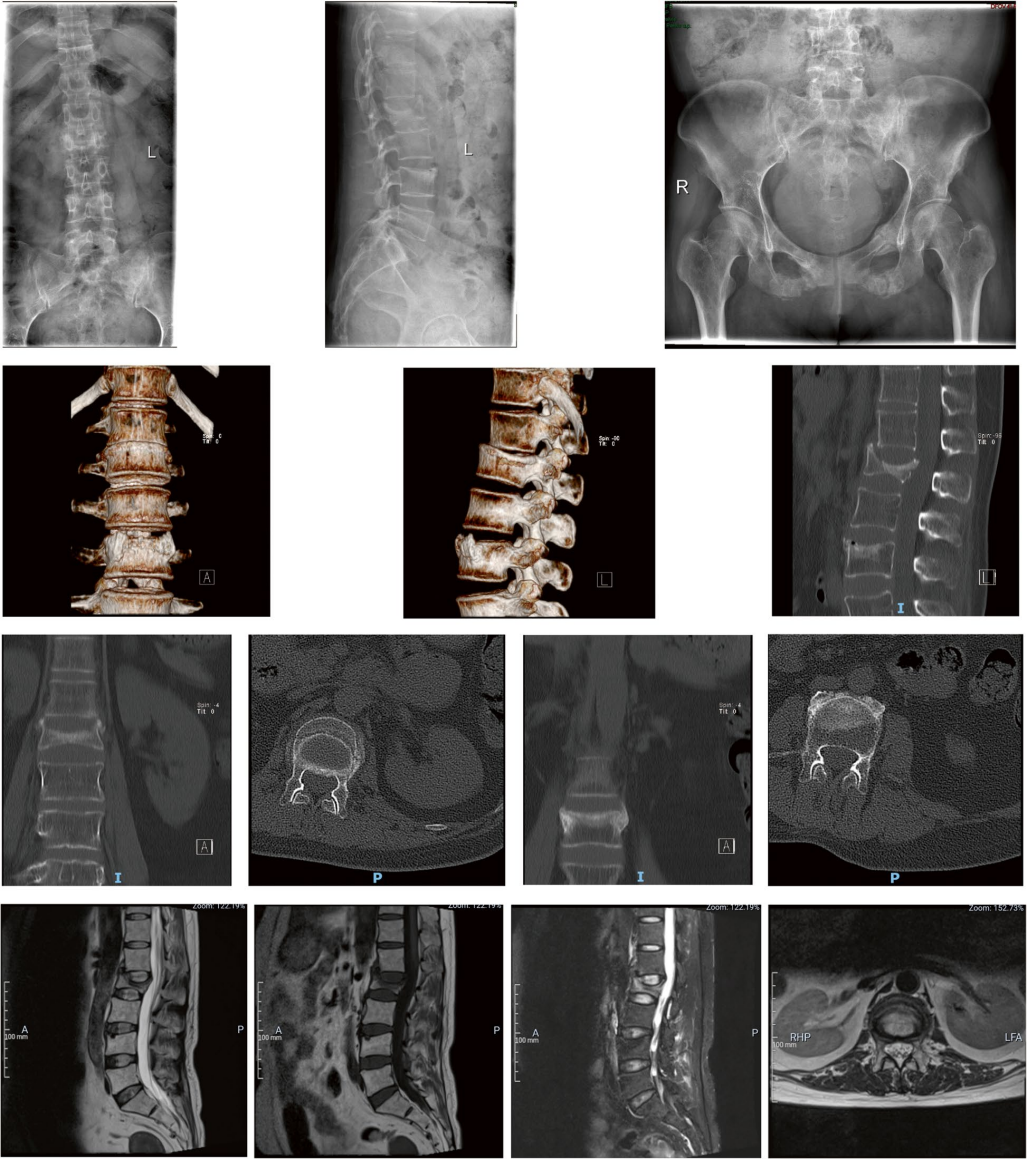
The imageological examinations showed fresh second vertebra fracture, old fourth lumbar vertebra fracture, and old pelvic fractures with callus formation on admission

**Fig. 2:**
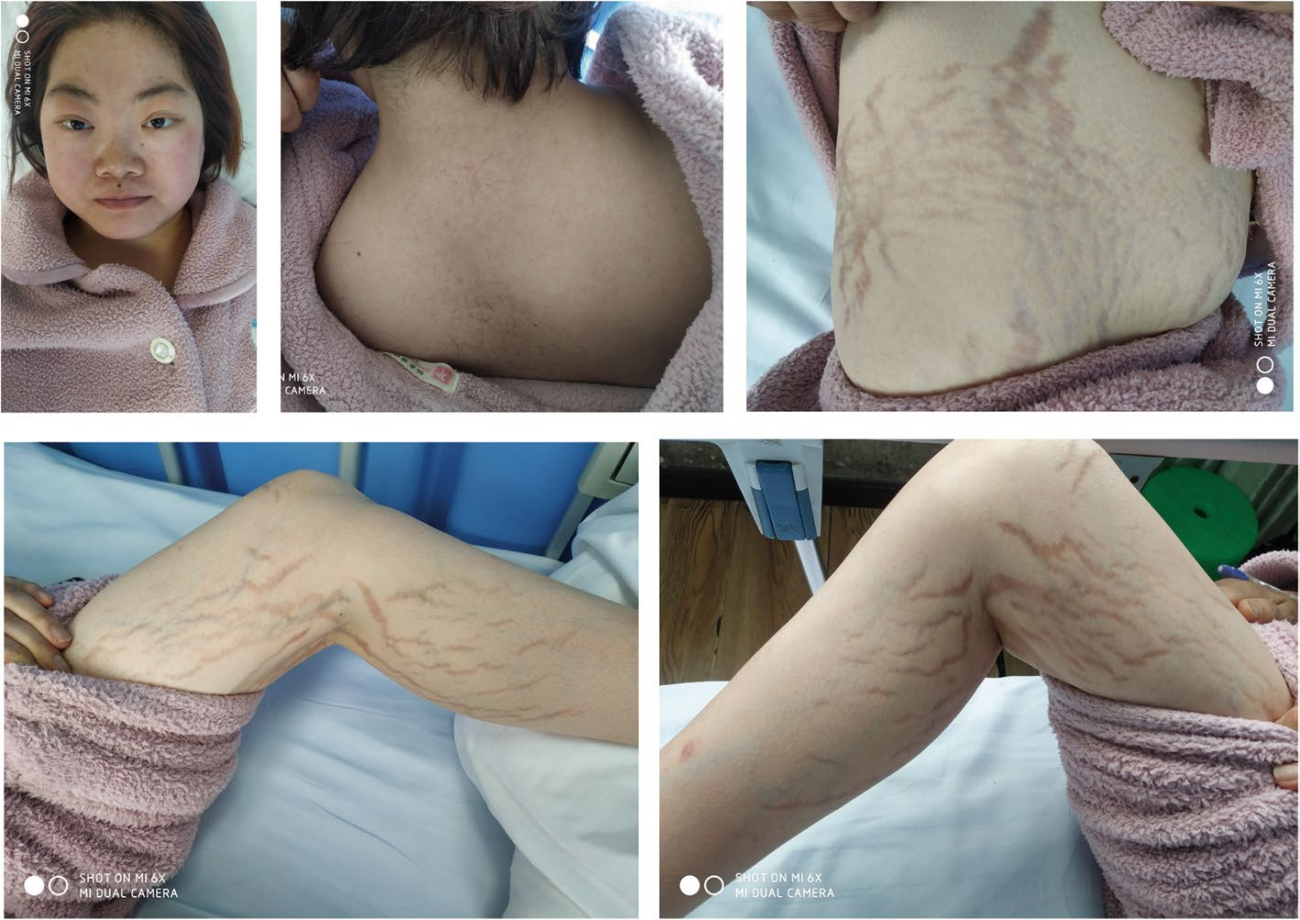
The patient’s appearance was plethoric moon face, buffalo hump, and purplish abdominal and thigh striae on admission

A 26-year-old woman visited the outpatient service of our institute for repeated low back pain with more than 1 year, aggravating for 2 months or so. The lumbar and pelvic X-ray showed second and fourth lumbar vertebra fractures and multiple pelvic fractures with callus formation (Fig. 1), then she was admitted to hospital for further treatment. The patients noted she gained weight 2 years ago, meanwhile, hypertension was found in periodical medical examination, and emphasized that she had no history of glucocorticoid abuse and trauma. The main manifestations of physical examination were plethoric moon face, buffalo hump, and purplish abdominal striae (Fig. 2). The magnetic resonance imaging (MRI) and computerized tomography (CT) of the lumbar spine confirmed that fresh fracture of second lumbar vertebra and old fracture of forth lumbar vertebra (Fig. 1), and dual energy X-ray absorptiometry (DXA) of lumbar spine revealed marked osteoporosis, in contrast, that of the femur was within the normal range (Table 1).

**Table 1:** Bone mineral density of lumbar spine and femur neck from dual energy X-ray absorptiometry

	ROI	BMD (g/cm^2^)	BMC (g)	area (cm^2^)	T-score	Z-score
Hip	Neck	0.581	2.71	4.66	−2.2 (− 28%)	−2.1 (− 28%)
	G.T	0.553	5.15	9.31	−1.2 (−18%)	−1.2 (− 18%)
	InterTro	0.865	15.24	17.62	−1.3 (− 18%)	−1.3 (− 17%)
	Overall hip joint	0.731	23.09	31.60	−1.3 (− 18%)	−1.3 (− 19%)
	Ward	0.492	0.39	0.79	NC	NC
Lumbar	L1	0.350	3.52	10.05	−4.9 (−60%)	−5.0 (− 60%)
	L2	0.546	6.66	12.19	−3.8 (−42%)	− 3.8 (− 42%)
	L3	0.509	7.32	14.38	−4.6 (− 49%)	−4.7 (− 49%)
	L4	0.534	9.12	17.08	−4.8 (− 49%)	−4.9 (− 49%)
	Total	0.496	26.62	53.70	−4.5 (− 49%)	−4.6 (− 49%)

On admission, her laboratory examinations were shown in Table 2, and the results demonstrated low levels of K+, Ca2+, and 25-hydroxyvitamin D, and high levels of alkaline phosphatase and parathyroid hormone (PTH), and so on. According to the above, Cushing’s syndrome was considered due to multiple fragility fractures, low BMD regarding age, and the gross findings. Then, free cortisol and adrenocorticotropic hormone (ACTH) in plasma were tested and the results showed 742.2 nmol/L and less than 1.00 pg/ml, respectively, indicating cortisol excess. To further confirm the diagnosis, a cortisol rhythm test was conducted. Consequently, plasma cortisol was 632.0 nmol/L at 8 am and 588.8 nmol/L at 24 pm, indicating excessive cortisol and disturbed rhythm (Table 3). Furthermore, a low-dose (1 mg) overnight dexamethasone suppression test was carried out, and the results showed that plasma cortisol the next morning was 812.9 nmol/L, indicating cortisol level was not suppressed (Table 4). Therefore, Cushing’s syndrome was clearly diagnosed in this patient.

**Table 2:** Biochemical laboratory data of the present patient

Names of index	At admission	Normal range
K^+^ (mmol/L)	2.6	3.5–5.3
Ca^2+^ (mmol/L)	2.09	2.2–2.7
Mg^2+^ (mmol/L)	1.01	0.7–1.0
Alkaline phosphatase (U/L)	292	35–100
Albumin (g/L)	39.3	40–55
International normalized ratio (INR)	0.84	0.85–1.50
Activated partial thromboplastin time (APTT)	23.00	23.3–32.5
Intact parathyroid hormone (pg/ml)	94.10	18.5–88.0
25-hydroxyvitamin D (ng/ml)	10.9	≥30

**Table 3:** The results of endocrinological examinations on admission

Names of index	At admission	Normal range
Random cortisol (nmol/L)	742.2	6–10 am:172–497; 4–8 pm:74–286
Random ACTH (pg/ml)	<1.00	7.2–63.3
Early morning cortisol (nmol/L)	632.0	6–10 am: 172–497; 4–8 pm: 74–286
Late evening cortisol (nmol/L)	588.8	6–10 am: 172–497; 4–8 pm: 74–286
Follicle-stimulating hormone (mIU/ml)	5.1	/
Luteinizing hormone (mIU/ml)	3.0	/
Prolactin (mIU/ml)	236.7	102–496
Sex hormone binding globulin (nmol/L)	20.67	32.4–128
Human growth factor (ng/ml)	0.08	0.126–9.88
Third generation thyrotropin (μIU/ml)	0.699	0.55–4.7
FT3 (pmol/L)	2.7	2.77–6.31
FT4 (pmol/L)	9.9	10.45–24.38
Aldosterone (ng/dl)	7.7	clinostatism: 1.0–16.0; erect position: 4.0–31.0

**Table 4:** ACTH and cortisol levels in various dexamethasone inhibition tests

Type of dexamethasone inhibition test	ACTH (pg/ml)	Cortisol (mmol/L)
Low dose	<1.00	812.9
High dose	<1.00	835.4

**Fig. 3:**
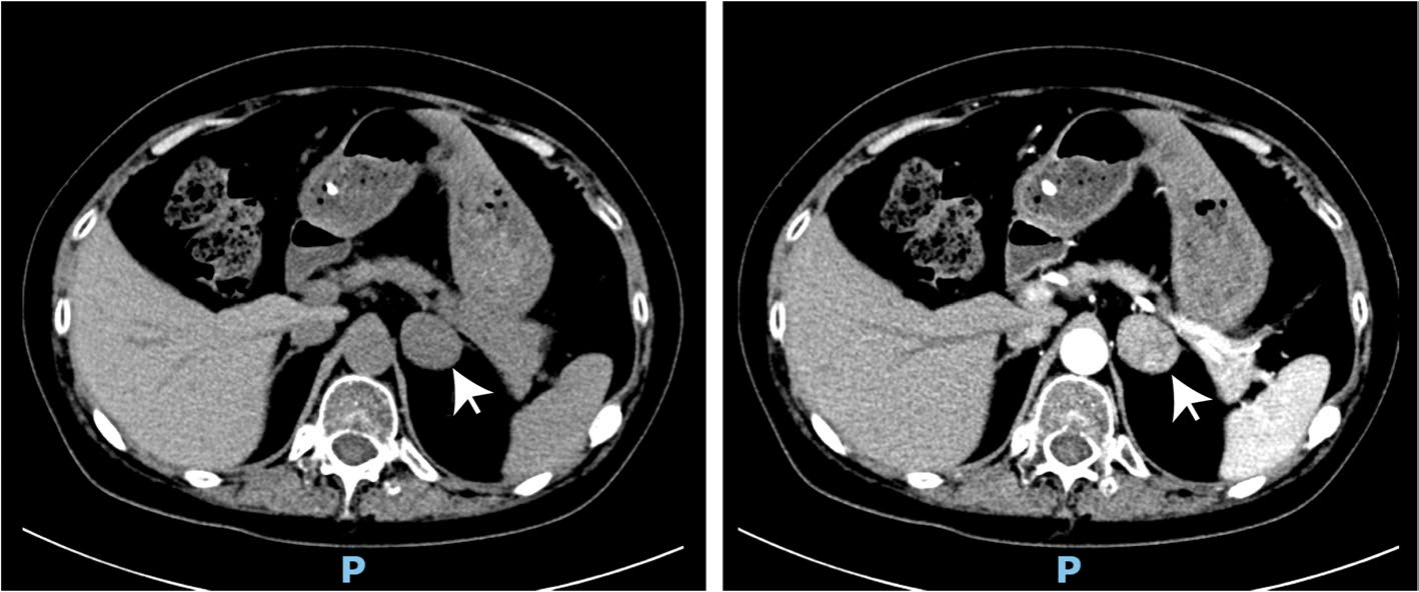
Adrenal gland CT showed a mass on left adrenal gland

After the diagnosis of Cushing’s syndrome, additional endocrinological examinations were performed to identify its etiology, as shown in Table 3. The hypophyseal hormones and gonadal hormones were almost all within normal limits, except for human growth hormone (HCG, 0.08 ng/ml) and sex hormone binding globulin (SHBG, 20.37 nmol/L), which were below normal values. In addition, thyroid stimulating hormone (TSH) was 0.707 μIU/mL, free T4 (FT4) was 9.9 pmol/L, and free T3 (FT3) was 2.7 pmol/L. Due to decreased level of HGH and disordered thyroid function, an MRI of pituitary gland and a high-dose dexamethasone suppression test were carried out. The results showed no abnormal pituitary gland, and cortisol levels were not suppressed more than 50% during a high-dose (8 mg) overnight dexamethasone suppression test (Table 4). Then, Cushing syndrome originated from adrenal gland was considered, and abdomen computed tomography was performed. Not unexpectedly, a tumor was observed on left adrenal gland (Fig. 3). The patient was referred to the urology department for further treatment of left adenoma. After receiving left adrenalectomy through laparoscope, the plasma ACTH and cortisol values returned to normal level on the first day postoperatively.

**Fig. 4:**
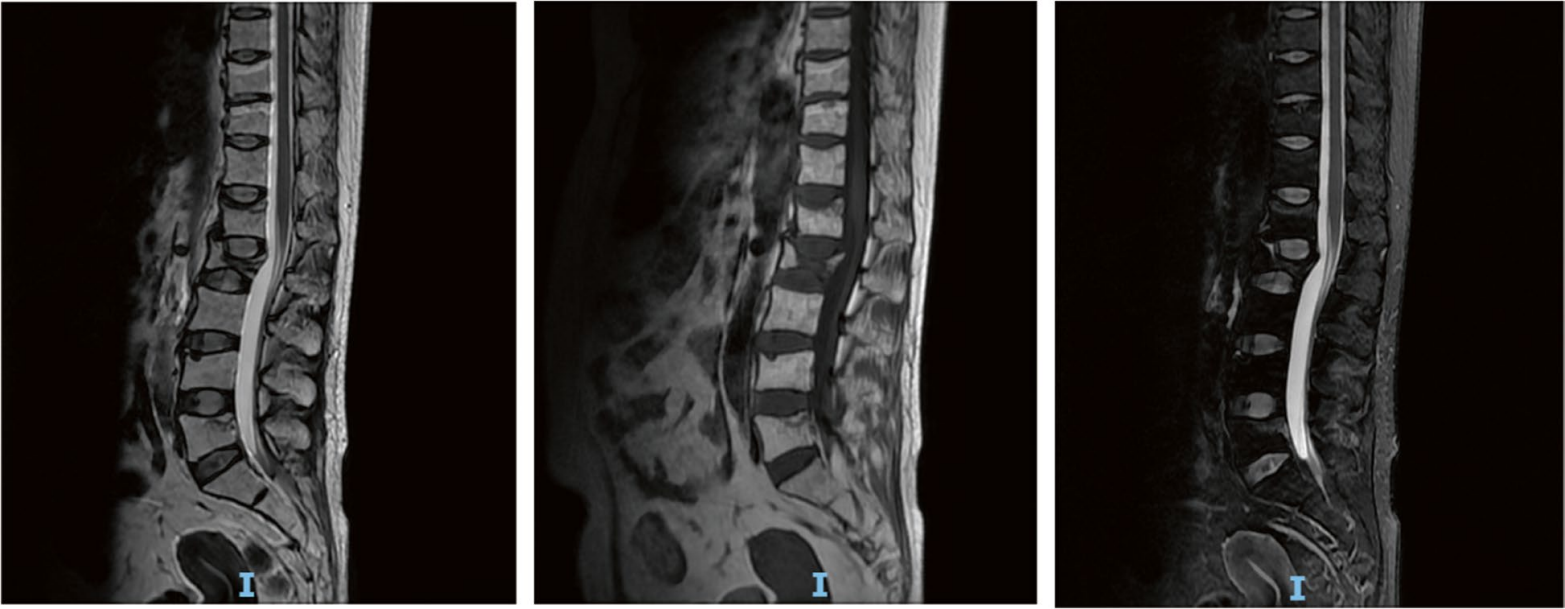
The MRI of lumbar spine showed the occurrence of new vertebral compression fracture in first lumbar vertebra duding hospitalization in the department of urology

Interestingly, during her hospitalization in the department of urology, the patient felt much more serious low back pian again, the reexamination MRI of lumbar spine showed the occurrence of new vertebral compression fracture in first lumbar vertebra (Fig. 4), but the patient refused operation for OVCF. Then, we recommended a series of standard conservative treatment regimens, including pain management, brace treatment, and anti-osteoporosis measures. The latter included minimum 700 mg of calcium daily through by supplementation, vitamin D supplements of at last 800 IU/day, and alendronate 10 mg/day for 6 months; salmon calcitonin nasal spray 200 IU/day for 2 months. Then, the patient was discharged for subsequent therapy. Three months after discharge, her pain was in complete remission without new onset of pain, and returned to normal life and work through telephone follow-up survey, but she refused to additional examinations, unfortunately, we don’t know the conditions of her bone mineral density and lumbar fractures.

## Discussion and conclusions

Chronic exposure to excess glucocorticoids brings about all sorts of manifestations of Cushing’s syndrome, with debilitating morbidities and increased mortality. Molecular mechanisms accounting for excess cortisol secretion by primary adrenal lesions and massive ACTH secretion from corticotroph or ectopic tumors have been identified [3, 4]. An estimated incidence of Cushing’s syndrome was 1.8/1,000,000 ⁓ 3.2/1,000,000 per year in various populations, and the age of diagnosis was 36 ⁓ 48 years with a significant female predominance (approximate female-to-male ratio: 3:1) [5–7]. Its manifestations vary from mild to rapid-onset serious versions, for example, fatigue, changes in adipose distribution (moon facies, buffalo hump or central adiposity), striae (purple color), thin skin, hypertension, fractures, and so on. Among them, the signs and symptoms of musculoskeletal system, such as, proximal muscle weakness, decreased bone mineral density (BMD), osteopenia, fractures, and low back pain, may be the first clinical manifestations [2].

Studies have described an impairment of bone status in 64 ⁓ 100% of patient with Cushing’s syndrome [7–11]. In particular, bone loss occurs in 40 ⁓ 178%, which is more frequent in patients with Cushing’s syndrome caused by adrenal tumors than that of pituitary tumors [12], osteoporosis occurs in 22 ⁓ 57%, and fractures occur in 11 ⁓ 76% of patients with Cushing’s syndrome, especially in thoracic and lumbar vertebrae and ribs, because trabecular bone is much more affected by glucocorticoid than cortical bone [10]. However, fractures can also occur rarely on long bone and pelvic bone, as reported in this case.

Noteworthy, vertebral fractures may be diagnosed in up to 75% of patients with Cushing’s syndrome, most of them being clinically associated with pain, functional limitations, and even height shortening by 3 ⁓ 10 cm of final stature [10]. For young patients with low-energy vertebral fractures, more attention must be paid on its etiology, because the principles of treatment regarding OVCF secondary to Cushing’s syndrome are different from that of primary osteoporosis. For such patients, the first and most important thing is etiological treatment to eliminate the predisposition of Cushing’s syndrome, such as, treatment of pituitary tumors or primary adrenal lesions. The treatment principles are detailed in other reviews of Cushing’s syndrome [13, 14].

Until now, the treatment of OVCF secondary to Cushing’s syndrome remains controversial. Percutaneous vertebroplasty (PVP) and kyphoplasty (PKP) are the main surgical options for primary osteoporosis associated compressed vertebrae fractures. PVP was first described in 1978 as a treatment for vertebral angioma and subsequently it has been used to treat both benign and malignant vertebral fractures. Then, vertebroplasty was introduced as an alternative option for alleviation of pain originated from OVCF. After that, this minimally invasive technique has gained widespread recognition, effectively alleviating pain and promoting functional recovery both in the short and long term [15, 16]. However, as with all other surgical procedures, PVP/PKP has its own indications and contraindications, and the main contraindications are fractures associated with neurological injury, and that fractures involve the posterior wall of vertebral bodies, leading to the high risk of bone cement leaking [17]. Although PVP/PKP could lead to rapid easement of pain and promptly functional rehabilitation, some patients may experience unexpected complications including new VCFs, spinal cord compression, nerve root injury, infection, and emboli [18]. The most in-depth studied complication is new VCFs, which can result in neurological deficit. The incidence of new VCFs after PVP or PKP was from 2.2 to 27.8% [19, 20]. Diverse risk factors of new VCFs have been identified, including lower BMD, cement distribution, intradiscal cement leakage, vertebrae height restoration, number of treated vertebrae, and so on [21–25]. After the injection of bone cement into the injured vertebrae, its stiffness changes accordingly, leading to increased stress between the cemented area and the non-cemented area (the same injured vertebrae or adjacent vertebrae), which is prone to re-fracture or new fracture. Beyond that, some researchers had reported that no beneficial effect of vertebroplasty as compared with a sham procedure in patients with osteoporotic vertebral fractures after treatment [26, 27] . In 2018, Cochrane Library reported a systematic review regarding of percutaneous vertebroplasty for osteoporotic vertebral compression fracture, and showed no demonstrable important clinical benefits compared with the placebo, and did not support a role for vertebroplasty to treat acute or subacute osteoporotic vertebral fractures in routine practice, based upon moderate to high quality evidences [28] . Therefore, PKP/PVP should be chosen carefully for patients with VCFs secondary to Cushing’s syndrome, but conservative treatments are recommended as a priority, including analgesic treatment, brace, and anti-osteoporosis treatment, and so on.

Anti-osteoporosis therapy is an important part in the treatment of all types of OVCF, including behavioral intervention and drug therapy. Unlike primary osteoporosis, etiological treatment combined with anti-osteoporosis treatment can reverse the loss of bone mass and increase bone density, to some extent, in osteoporosis resulted from Cushing’s syndrome [29, 30]. Lifestyle approaches, for example, strengthening resistance exercise, fall interventions, reducing alcohol intake, smoking cessation, and adequate supplementation calcium and vitamin D, play key roles in improving musculoskeletal status at any time [31–33]. Among them, adequate dietary intakes of key bone nutrients, such as, calcium and vitamin D contribute to bone health. A meta-analysis, by Tang et al. [34], supported that supplementation calcium or combination with vitamin D could prevent osteoporosis in population aged 50 years or older, whereafter, the high-level evidences of evidence-based medicine also suggested that supplementation with both calcium and vitamin D could reduce hip and vertebral fractures [35, 36]. Therefore, a balanced, nutritious diet is strongly recommended in postmenopausal women and men age more than 50 years. A minimum 700 mg/day calcium and at least 800 IU/day vitamin D through dietary intake or by supplementation was strongly recommended by the National Osteoporosis Guideline Group [37].

The main pharmacological therapies for osteoporosis are antiresorptive and anabolic drugs, effectively reducing fracture risk [38]. As we known, osteoporosis derives from an imbalance between bone resorption and bone formation. Antiresorptive drugs can reduce the number and lifespan of osteoclasts, meanwhile, restrain its activity, as a result, increase bone mass and decreases the chance of vertebral and non-vertebral fractures. These drugs include bisphosphonates, oestrogen and selective oestrogen receptor-modulating drugs, strontium ranelate and receptor activator of NF kappa B ligand inhibitor. A randomized trail confirmed the beneficial effect of alendronate on the risk of vertebral fractures among women with low bone mass and existing vertebral fractures [39]. And, alendronate also obviously increases BMD and prevent vertebral fractures in men with osteoporosis [40]. Furthermore, alendronate can increase bone density and decrease vertebral fractures in patients with osteoporosis induced by glucocorticoid therapy [41]. In addition, risedronate and zoledronate have proven their ability to decrease the risk of vertebral and non-vertebral fractures in postmenopausal women and men with osteoporosis [42–44]. In 2022, the UK National Osteoporosis Guideline Group suggested that anti-resorptive therapy was the first-line option for osteoporosis and oral bisphosphonates or intravenous zoledronate was strongly recommended to people with risk of fragility fractures [37]. Anabolic drugs primarily recruit and activate osteoblast, further stimulating bone formation, including teriparatide, abaloparatide, and romosozumab. Teriparatide, a recombinant parathyroid hormone identical to the 34 N-terminal amino acids of human PTH, increases osteoblast recruitment and activity to promote bone formation. Studies reported that teriparatide could linearly increase BMD of spine, but not that of proximal femora [45], reduce the risk of new vertebral and non-vertebral fractures in women with osteoporosis for 21 months treatment [46], and also decrease worsening or new back pain. In patients with very high fracture risk, especially with vertebral fractures, teriparatide or romosozumab is usually recommended as an alternative option for anti-osteoporosis treatment [37].

Previous studies have reported that osteoporosis secondary to excessive glucocorticoid is reversible [47, 48]. Although no relevant changes in BMD six months after Cushing’s syndrome cure, but the osteoblast activity was restored based on elevated osteocalcin levels, and remarkable improvement on BMD can be observed within 12–36 months after the cortisol returned to normal lev. Akiko Kawamata et al. [30] reported that significant improvement in BMD, particularly in the lumbar spine, had been achieved followed by operative treatment of hypercortisolism in patients with Cushing’s syndrome due to adrenal adenoma. However, bone mass increases very slowly and usually takes 10 years to return to normal [49]. Thus, anti-resorptive drugs would be beneficial to patients with osteoporosis since they are more likely to develop fractures. Studies reported that alendronate demonstrated advantageous effects on BMD of lumbar spine and hip in patients with glucocorticoids therapy [41, 50, 51]. Saag KG et al. [52] found that obvious increase of BMD and less fractures were found in group with 20 μg of parathyroid hormone treatment per day instead of that with daily administration of 10 mg alendronate at 6th and 12th month. Recent consensus regarding the treatment of glucocorticoid-induced osteoporosis is that oral bisphosphonates are regarded as first-line option in most patients by reason of its better cost performance and good safety. Furthermore, teriparatide could be considered as an alternative option in patients at greater risk of fractures, based on its superiority in effect on BMD and vertebral fracture risk. In addition, it is necessary to supplement calcium and vitamin D in due time [53, 54].

In this case, while in hospital, analgesics (paracetamol and dihydrocodeine tartrate tablets) and bracing therapy were given to the patient, subsequently, the visual analogue scale (VAS) scores of low back pain were decreased from 7 to 3, then the patient refused operation for OVCF and chose conservative treatments. In addition to analgesics and bracing treatments, an anti-osteoporosis regimen was given to her: minimum 700 mg of calcium daily through by supplementation, vitamin D supplements of at last 800 IU/day, and alendronate 10 mg/day for 6 months; salmon calcitonin nasal spray 200 IU/day for 2 months. After three months of conservative treatment, the patient reported no obvious low back pain and returned to normal life and work by telephone follow-up survey. Therefore, for young patients with non-violent vertebral fractures, especially in women, it is necessary for us to consider the possibility of Cushing’s syndrome, and the main principle of treatment is to identify and eliminate etiology. In term of OVCF secondary to Cushing’s syndrome without neurological damage, we prefer systematic conservative treatment, including pain managements, brace treatment, and anti-osteoporosis measures, to surgical treatment. Among them, anti-osteoporosis treatment has the highest priority because of the reversibility of osteoporosis caused by Cushing’s syndrome.

## Data Availability

All data generated or analyzed during this study included in this published article.

## Abbreviations

OVCF: Osteoporotic vertebral compression fracture
GS: Cushing’s syndrome
BMD: Bone mineral density
MRI: Magnetic resonance imaging
CT: Computerized tomography
DXA: Dual energy X-ray absorptiometry
PTH: Parathyroid hormone
ACTH: Adrenocorticotropic hormone
HGG: Human growth hormone
SHBG: Sex hormone binding globulin
TSH: Thyroid stimulating hormone
PVP: Percutaneous vertebroplasty
PKP: Percutaneous kyphoplasty
VAS: Visual analogue scale
